# Targeting Liver Cancer Stem Cells: An Alternative Therapeutic Approach for Liver Cancer

**DOI:** 10.3390/cancers12102746

**Published:** 2020-09-24

**Authors:** Hwa-Yong Lee, In-Sun Hong

**Affiliations:** 1 Department of Biomedical Science, Jungwon University, 85 Goesan-eup, Munmu-ro, Goesan-gun, Chungcheongbuk-do 367700, Korea; hylee@jwu.ac.kr; 2 Department of Health Sciences and Technology, GAIHST, Gachon University, Incheon 21999, Korea; 3 Department of Molecular Medicine, School of Medicine, Gachon University, Incheon 406840, Korea

**Keywords:** hepatic cancer stem cells, ALDH, CD133, EpCAM, Wnt/β-catenin signaling, notch signaling

## Abstract

**Simple Summary:**

Cancer stem cells (CSCs) are known to be highly resistant to conventional therapeutic approaches, such as chemotherapeutic drugs and radiation. Therefore, selectively targeting CSCs with specific markers or signaling pathways can be an effective therapeutic strategy for treating chemotherapy-resistant liver cancer. However, there is not enough information currently available to make a conclusive statement regarding hepatic CSC-specific signaling pathways and biomarkers. In present study, we provide an overview of the current knowledge on the specific surface markers and critical signaling pathways of hepatic CSC.

**Abstract:**

The first report of cancer stem cell (CSC) from Bruce et al. has demonstrated the relatively rare population of stem-like cells in acute myeloid leukemia (AML). The discovery of leukemic CSCs prompted further identification of CSCs in multiple types of solid tumor. Recently, extensive research has attempted to identity CSCs in multiple types of solid tumors in the brain, colon, head and neck, liver, and lung. Based on these studies, we hypothesize that the initiation and progression of most malignant tumors rely largely on the CSC population. Recent studies indicated that stem cell-related markers or signaling pathways, such as aldehyde dehydrogenase (ALDH), CD133, epithelial cell adhesion molecule (EpCAM), Wnt/β-catenin signaling, and Notch signaling, contribute to the initiation and progression of various liver cancer types. Importantly, CSCs are markedly resistant to conventional therapeutic approaches and current targeted therapeutics. Therefore, it is believed that selectively targeting specific markers and/or signaling pathways of hepatic CSCs is an effective therapeutic strategy for treating chemotherapy-resistant liver cancer. Here, we provide an overview of the current knowledge on the hepatic CSC hypothesis and discuss the specific surface markers and critical signaling pathways involved in the development and maintenance of hepatic CSC subpopulations.

## 1. Introduction

Liver cancer is the sixth most frequently diagnosed solid tumor worldwide in 2018 [1] and the third leading cause of cancer-related deaths [2]. Cancer that begins in the liver is called primary liver cancer. Hepatocellular carcinoma (HCC) represents the predominant histological subtype and accounts for approximately 80% of all primary liver cancer patients [3]. Intrahepatic cholangiocarcinoma (ICC) is the second most common primary liver cancer, representing approximately 20% of patients [4]. Both HCC and ICC are extremely heterogeneous tumors at both the genetic and phenotypic level. A newly defined mixed or combined hepatocellular carcinoma-cholangiocarcinoma (HCC-CC) characterized by dual hepatocellular and biliary epithelial differentiation suggests the existence of bipotent hepatic stem/progenitor cells with both hepatocyte and cholangiocyte lineages [5]. Indeed, recent studies indicate that HCC, ICC, and HCC-CC are highly heterogeneous in terms of their cellular and molecular characteristics and contain a small subset of self-renewing cells preferentially expressing various stem cell markers [6,7,8,9].

Furthermore, several studies have shown that purified CD133^+^ cells from HCC cell lines have higher proliferation potential and tumorigenic ability in animal models and exhibit stem cell-like characteristics, including their ability to self-renew and differentiate into multiple cell lineages [10]. Moreover, a subset of ICCs expresses stem/progenitor cell-related markers, suggesting CSCs are a possible cell source for ICC [11,12,13,14]. Thus, identifying and selectively targeting CSCs represents a feasible therapeutic strategy for treating liver cancer regardless of the underlying cause. However, there is not enough information currently available to make a conclusive statement regarding the cellular origin of hepatocarcinogenesis, and additional characteristics related to hepatic CSC-specific signaling pathways and markers remain to be elucidated.

## 2. The Origin of Cancer Stem Cells

Owing to the similarities between normal stem cells and CSCs for instance the capacity to self-renew and multi-lineage differentiation [15], many recent investigations have sought to determine whether CSCs arise from the dysregulated normal stem cells or more differentiated cells through multiple mutations. The answer may largely depend on the specific types of cancers and malignant phenotypes. The origin of CSCs is still under debate for the past few years [15,16]. Somatic stem cells are able to divide indefinitely and differentiate into some or all cell types of the tissue or organ [17]. In fact, it has been postulated that CSCs might originate from cells with stem-like characteristics or from normal stem cells by the accumulation of multiple mutations that render the stem cells cancerous [18]. Leukemic stem cells share several properties with normal hematopoietic stem cells (HSCs), supporting the stem-cell origin hypothesis [19,20]. Stem cells are usually characterized by their ability to undergo unlimited self-renewing cell division. It is therefore reasonable to hypothesize that these extended lifespan of a stem cells makes it a prime target for the multiple mutations necessary for tumor progression [21]. However, this hypothesis probably demands high mutation rates, because few somatic stem cells exist naturally in the adult tissues. Besides the stem-cell origin hypothesis, recent publications have suggested that cancer cells can also derive from fully differentiated (or “mature”) cells by undergoing de-differentiation to become more stem cell-like characteristics [22,23]. In this hypothesis, tumorigenesis is initiated by oncogenic mutations in a differentiated cell and subsequent acquisition of stem-cell-like features functions through a process of de-differentiation. Probability, the more differentiated cells exist in adult tissues, the greater the chance of mutations that can cause oncogenic transformation [24]. Surprisingly, the entire sequence of tumorigenesis can be mediated by only few steps; Takahashi et al. have recently revealed that terminally differentiated adult fibroblasts can be genetically “re-programmed” into induced pluripotent stem (iPS) cells by introducing only four transcription factors (Myc, Oct4, Sox2, and Klf4) [25,26]. However, currently there is not enough information available to make a conclusive statement regarding the origin of CSCs, and further investigation is warranted.

## 3. Cancer Stem Cells: Implications for Hepatocarcinogenesis

### 3.1. Identification of CSCs in Various Types of Tumors

The majority of cells in bulk tumors have limited tumorigenic growth and self-renewal potential; indeed, only a small population of tumor cells possess a marked self-renewal capacity, and differentiation and the ability to generate new tumors [27]. These higher tumorigenic subpopulations are known as CSCs holding a higher tumorigenic potential [28]. The CSC concept has been proposed to explain the high degree of phenotypic and functional heterogeneity of cancer cells within a given tumor [21]. In the 1960s, Bruce et al. demonstrated that only small fractions (1–4%) of leukemic cells can form colonies in in vitro and initiate new tumors in recipient animals [29]. The identification of leukemic CSCs prompted further studies to identify and isolate CSCs in various solid tumors. Extensive research in the past few decades has identified CSCs in multiple solid tumors, including colon [30], brain [31], lung [32], liver [33], and other cancers [34]. CSCs are generally defined by their distinct and specific surface antigen expression [35,36,37] and by their capacity to generate spherical colonies from single cell in suspension cultures [38]. Moreover, CSCs exhibit a higher resistance to standard chemotherapy [39] and radiation therapy [40] through deregulated apoptosis and survival signaling. These drug-resistance properties of CSCs suggest that the majority of standard therapeutic approaches can eliminate the bulk tumor cells but may ultimately fail to obtain reliable clinical responses because conventional treatments are not as effective at eliminating CSCs; thus, the remaining CSCs are able to re-initiate tumor development in patients.

### 3.2. CSCs as a Novel Therapeutic Target

**Figure 1:**
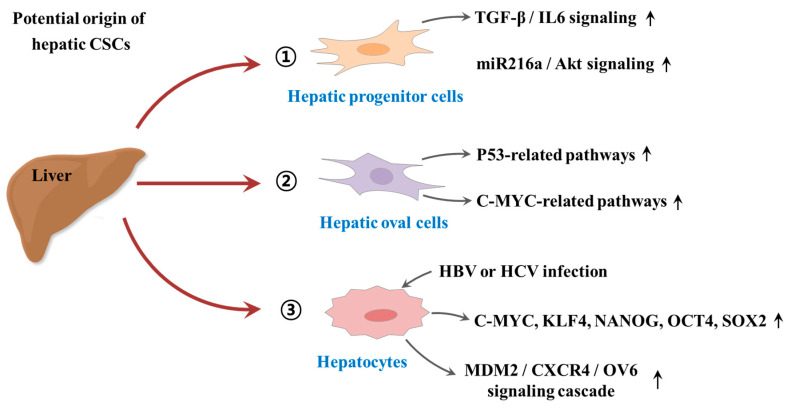
Schematic diagram summarizing the potential origin of hepatic cancer stem cells (CSCs). Hepatic progenitor cells can be transformed into tumor-initiating cells by activation of transforming growth factor beta (TGFβ)/interleukin-6 (IL-6)-related signaling and miR216a/Akt-dependent signaling pathways. Hepatic oval cells (HOCs) were involved carcinogenesis of hepatocellular carcinoma (HCC) through p53 and c-myc related signaling pathways. In addition, the infection of hepatitis B or C virus (HBV or HCV) significantly increases the malignant transformation into HCC by enhancing the expressions of CSCs-associated transcription factors or activating MDM2/CXCR4/OV6 signaling cascades. “↑” means increase.

Despite some promising therapeutic outcomes, conventional therapeutic approaches against tumors have many limitations that frequently lead to local recurrence with subsequent metastasis and poor survival. The main reason for these cancer relapse and unsatisfactory long-term clinical responses is resistance to conventional therapy. CSC-mediated multiple drug resistance has been observed over the past half-century in various tumor types, including leukemia [41], colorectal [42], brain [43], pancreatic [44], melanoma [45], breast [46], and cancers. Moreover, CSC-mediated radioresistance was also observed in breast [47] and brain [48] cancers. Over the past years, many efforts have been devoted to investigate the potential origin of hepatic CSCs. For instance, Tang et al. found that hepatic progenitor cells can be transformed into tumor-initiating cells by transforming growth factor beta (TGFβ) and interleukin-6 (IL-6)-related signaling pathways [49]. Consistently, Wu et al. also revealed that a small subset of hepatic progenitor cells express tumor initiating cell markers during hepatocarcinogenesis in both rat and human models, and they are transformed through miR216a and Akt-dependent pathway [50]. Another study also investigated pathological characteristics of hepatic oval cells (HOCs) and their potential roles during the progression of HCC [51]. Dumble et al. showed that HOCs were involved carcinogenesis of HCC through p53 signaling pathway [52]. Likewise, c-myc expression may promote the hepatocarcinogenesis of HOCs [53]. In addition, the infection of hepatitis B or C virus (HBV or HCV) significantly increases the malignant transformation into HCC by approximately 15-to 20-fold compared with HCV-negative subjects [54]. HBV infection facilitates the expressions of various CSCs-associated transcription factors (c-Myc, Klf4, Nanog, Oct4, and Sox2) and CSCs-related genes (CD90, CD117, and CD133), and thus stimulates the self-renewal capacity of hepatocyte derived cells [55]. Similarly, Wang et al. also found that the overexpression of hepatitis B virus X protein enhances the stem-like properties and tumorigenic potential of OV positive liver CSCs by activating the MDM2/CXCR4/OV6 signaling cascades [56]. In this context, the development of novel therapeutic strategies that selectively eliminate CSCs and leave the normal and healthy cells largely unaffected is urgently required. An improvement can potentially be achieved by the selective targeting of subtle differences in surface antigens regulating their functions as well as alterations in signaling pathways of CSCs. Since their identification in multiple solid tumors and leukemia, various CSC elimination strategies selectively targeting CSC-specific surface markers and signaling pathways have been applied. While most are still at the preclinical stage, currently, some of these strategies can successfully eliminate CSCs and thereby prevent local recurrence with subsequent metastasis. The potential origin of hepatic CSCs is summarized in Figure 1.

### 3.3. The Hepatic CSC Microenvironment

Hepatic cancer occurs more frequently in patients with chronic liver diseases due to the chronic inflammatory response and continuous hepatocyte destruction/regeneration that occurs [57]. A variety of physiological changes that take place during long-term liver regeneration and inflammation can enhance both the initiation and promotion phases of hepatocarcinogenesis. These changes accelerate the accumulation of genome instability through genetic and epigenetic alterations, expansion of resident hepatic stem/progenitor cell populations, and modification of the hepatic microenvironment. Chronic liver diseases can also induce proliferation of hepatic stem/progenitor cells [58]. Their recruitment, proliferation, and development are tightly regulated by various factors transmitted from stem cell niches referred to as a specialized microenvironment [59]. The liver microenvironment is drastically changed in chronic liver diseases to favor tumors, including increased expansion of hepatic progenitor cells and endothelial progenitor cells, hepatic infiltration by lymphocytes, and the activation of hepatic stellate cells. During chronic liver diseases, hepatic stellate cells are activated and proliferate, which results in scar formation and fibrosis with excessive extracellular matrix (ECM) deposition [60]. These dynamic physiological conditions can cooperatively affect liver tumorigenesis by supporting hepatic CSC development. For instance, compared with normal fibroblasts, cancer-associated fibroblasts (CAFs) have enhanced self-renewal capacity and increased secretion of various growth factors, such as CXCL12, hepatocyte growth factor (HGF), platelet-derived growth factor (PDGF), and vascular endothelial growth factor (VEGF), which can promote tumorigenesis [61]. Multiple growth factors or cytokines secreted by endothelial cells (ECs) and CSCs in the tumor microenvironment can promote the transformation of normal fibroblasts into CAFs [62]. Subsequently, transformed CAFs can stimulate the stem-like properties of hepatic CSCs by modulating autophagy [63]. Similarly, myofibroblast activation releases several growth factors and cytokines that may result in sustained tumor progression [64]. Mesenchymal stem cells (MSCs) have been implicated in promoting cancer cell growth, invasion/metastasis, vasculogenesis, and immunosuppression within tumor microenvironment for the restoration of cancer stem cells [65,66] by secreting various growth factors, cytokines, chemokines, and ECM components [67]. Indeed, Mi et al. found that considerable amount of IL-6 was secreted by MSCs and subsequently promoted human HCC invasion by activating IL-6/STAT3 signaling pathway [68]. In addition, the tumor microenvironment is characterized by chronic inflammatory conditions, which can promote tumor cell growth, survival, invasion, and metastasis [69]. Lymphocytic infiltration can cause the release of inflammatory molecules and the formation of oxygen free radicals, which results in DNA damage and other stresses that can stimulate tumor growth [70].

### 3.4. The Effect of Chemotherapy/Radiotherapy on Hepatic CSCs

**Figure 2:**
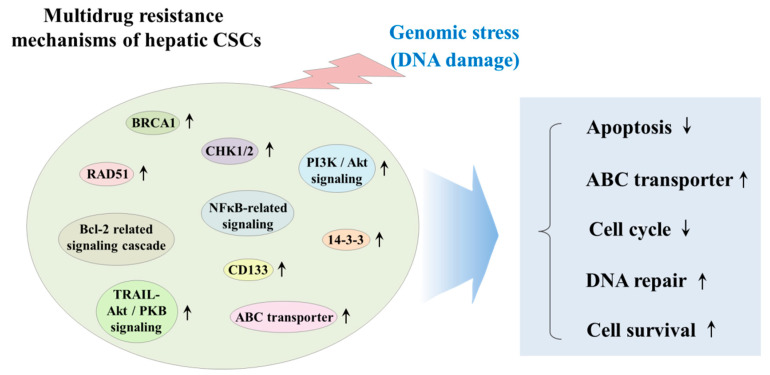
Schematic diagram summarizing the multidrug resistance mechanisms of hepatic CSCs. CSCs have been known to exhibit various genetic and/or epigenetic alternations, which are related to the resistance to classical therapeutic strategies, such as chemotherapy and radiotherapy. These various alternations include dysregulation of ATP-binding cassette (ABC) membrane transporters, cell cycle arrest (quiescent state), enhanced DNA repair efficiency, and high resistance to anticancer drug-induced apoptosis. “↑” means increase; “↓” means decrease.

CSCs have been known to exhibit various genetic and/or epigenetic alternations that are associated with the resistance to classical therapeutic strategies, such as chemotherapy and radiotherapy [71]. These various alternations include dysregulation of ATP-binding cassette (ABC) membrane transporters, cell cycle arrest (quiescent state), enhanced DNA repair efficiency, and high resistance to anticancer drug-induced apoptosis [72]. Radiation and many types of chemotherapeutic agents exert their anticancer effects by inducing DNA damage to cancer cells; thus, it seems reasonable to hypothesize that the resistance of CSCs to classical therapeutic approaches may be due to the increased expression of DNA repair-related genes, such as BRCA1 and RAD51 [73]. One of the most potent regulators of CSC resistance to DNA damaging chemotherapeutic drugs is DNA damage checkpoint protein kinases (CHKs), which are activated by genotoxic stress and delay the cell cycle progression to facilitate DNA repair [74]. Lee et al. found that depletion of 14-3-3ζ, which regulates cell cycle, differentiation, and apoptosis, increases the sensitivity to radiation therapy in CD133^+^ Huh7 liver cancer stem cells [75]. Ma et al. reported that CD133^+^ hepatic CSCs exhibit greater chemoresistance than CD133^−^ subpopulation by activating well-known pro-survival Akt/PKB and anti-apoptotic Bcl-2 signaling pathways [76]. Another important regulator of the DNA repair systems against both endogenous and exogenous sources of DNA damage in stem cells is ATP-binding cassette transporters (ABC transporters), which can selectively extrude various toxic substrates, leading to multidrug resistance (MDR) [77]. Indeed, Fung et al. found that enhanced expression levels of ABC transporters significantly promote chemoresistance, epithelial–mesenchymal transition (EMT) and cancer stemness in HCC model [78]. PI3K/Akt, which is one of the most potent prosurvival signaling pathways, contributes to the maintenance and survival and also triggers endogenous drug resistance in CSCs [79]. Indeed, Kahraman et al. showed PI3K/Akt/mTOR pathway-mediated resistance to Rapamycin to Sorafenib cotreatment in CD133^+^/EpCAM^+^ hepetic CSCs [80]. Tumor necrosis factor (TNF)-related apoptosis-inducing ligand (TRAIL) plays an important role in cancer therapy by inducing selective apoptosis of cancer cells while having little effect on the normal cells [81]. Zhu et al. reveal that TRAIL mediates drug resistance in various hepatic CSC models (PLC, HepG2 and Huh7 LC cells) through PI3K/Akt/Bad signaling cascades [82]. Another promising target molecular to induce apoptosis in CSCs is nuclear factor kappa B (NFκB), which is known as an antiapoptotic signal transcription factor, can be activated by various chemodrugs including sorafenib [83]. Zou et al. showed that sorafenib-induced NF-κB activation contributes to the enhanced resistance to sorafenib in CD133-positive sphere-forming hepatic CSCs [84]. The multidrug resistance mechanisms of hepatic CSCs are summarized in Figure 2.

## 4. Surface Marker-Based Therapies

### 4.1. Aldehyde Dehydrogenase (ALDHs)

Aldehyde dehydrogenases (ALDHs) are a superfamily of oxidizing enzymes that catalyze the oxidation (dehydrogenation) of various aldehyde derivatives. These proteins were first described as enzymes conferring resistance against cyclophosphamide in in stem cells and cancer [85]. In cancer cells, ALDH functions as a retinal dehydrogenase (RALDH) that is involved in a metabolic process of converting retinol (also known as vitamin A) into active ligand retinoic acid (RA) [86]. Retinoic acids are known to play many roles in the regulation of major embryonic growth and patterning decisions. ALDHs are highly expressed in primitive hematopoietic progenitors and multipotent neuronal stem cells [85]. Recent studies have demonstrated that ALDH^high^ cancer cells enhanced tumorigenic capacity and chemotherapeutic drug resistance in various types of cancer [87]. Indeed, a subpopulation of cells with high ALDH activity has been observed in the highly tumorigenic colon CSCs with a stem-like EpCAM^high^/CD44^+^ phenotype [88]. Moreover, high ALDH activity in breast CSCs correlates with more aggressive tumor behavior as well as chemoresistance, and thus may be used as a poor clinical outcome [89]. Ma et al. have found that ALDH-positive cells highly expressed primitive cell surface marker CD133 and ALDH could be used as a positive marker for tumorigenic HCC CSCs [90]. Ma et al. discovered in their purified subpopulations that CD133^+^ALDH^+^ cells are more tumorigenic than CD133^+^ALDH^−^ cells when grafted to mice [90]. In this context, it is reasonable to assume that ALDH may serve as a novel marker of poor prognosis and potential therapeutic target for the treatment of hepatocellular carcinoma [91]. Silencing snail expression significantly inhibits ALDH1 expression and subsequently suppresses stem-like properties and in vivo tumorigenic activities of CD44^+^CD24^−^ALDH1^+^ cells [92].

### 4.2. EpCAM

Epithelial cell adhesion molecule (EpCAM, CD326) is a transmembrane glycoprotein that mediates Ca-independent cell–cell adhesion in epithelial cells [93]. EpCAM was originally identified as a cell–cell adhesion molecule [94,95]; however, it is a new type of cell adhesion molecule (CAM) that does not structurally resemble any of the four major adhesion molecule families (cadherins, CAMs, integrins, and selectins). EpCAM was initially described as a dominant tumor-associated antigen in colon cancer cells [96] and is involved in the maintenance of stem-cell phenotypes [97] and malignant tumor characteristics [98]. Recently, a number of researchers have focused on the regulatory role of EpCAM in hepatic carcinogenesis [99,100,101]. Following a microarray analysis on hepatic tumor tissue samples, EpCAM^+^ subpopulations exhibited significantly high levels of stem cell-related markers [98]. Moreover, EpCAM^+^ subpopulations were more tumorigenic than their EpCAM^−^ counterparts in nude mice [102]. These data suggest that EpCAM could be a potential target for the diagnosis and therapy of hepatic cancers. Yamashita et al. demonstrated that activation of the Wnt/β-catenin signaling pathway stimulates EpCAM expression which, in turn, negatively affects the prognosis of HCC patients [103,104]. Wang et al. [105] and Arzumanyan et al. [106] demonstrated that hepatitis B antigen HBx promotes the self-renewal and tumorigenicity of EpCAM^+^ hepatic progenitor cells by stimulating β-catenin signaling and miR-181 expression. Ji et al. also demonstrates that aberrant expression of long noncoding RNA, LINC00152 increases EpCAM expression, resulting in enhanced growth of HCC both in vitro and in vivo by promoting Akt/mTOR signaling cascade [107].

### 4.3. Side Population

Stem cells with relatively high expression of ATP-binding cassette (ABC) transporter superfamily members show resistance against the absorption of unrelated (toxic) substances by pumping these compounds across cell membranes [108]. This action results in a low Hoechst 33342 side population (SP) [109]. In 2006, Chiba et al. successfully used Hoechst dye 33342-effluxing SP cells to identify hepatic cancer cells with stem-like properties in HCC [110]. Among the four HCC cell lines analyzed, SP fractions were detected in two cell lines as a minority population consisting of approximately less than 1% of the total cell population. This subset of cells was characterized with a higher tumorigenic potential when compared with non-SP counterparts. Importantly, tumor formation occurred following the injection of as few as 1 × 10^3^ SP-derived cells in NOD/SCID mice, and a high tumorigenic rate was maintained indefinitely upon serial transplantation in vivo; in contrast, as many as 1 × 10^6^ non-SP cells were not sufficient to initiate measurable tumor formation [110]. The relevance of the SP phenotype for tumorigenic potential and as a potential marker of CSCs suggests an urgent need for the development of effective SP-targeted therapeutic strategies for the treatment of liver cancer. Hu et al. discovered that Akt signaling was able to enhance the efflux activity of side population (SP) cells via altering the subcellular localization and distribution of ABC transporter (known to confer drug resistance) in HCC cell line MHCC-97L [111]. Moreover, Park et al. demonstrated that IL-8 increased drug resistance through SP enrichment as well as enhanced multidrug resistance 1 (MDR-1) expression [112]. Chiba et al. described an important regulatory role for BMI1 in the self-renewal ability and enrichment of tumorigenic stem-like SP cells in HCC. They suggested that BMI1 might be a potential therapeutic target for the elimination of tumor-initiating SP fractions in HCC [113].

### 4.4. CD44

CD44 is a single-chain transmembrane receptor for hyaluronic acid (HA) and has also recently been recognized as a marker for CSCs from various solid tumors, including gastric [114], bladder [115], pancreatic [37], cervical [116], lung [117], colon [118], ovarian [119], breast [120], and prostate cancers [121]. In hepatic cancers, CD44 has been extensively used in combination with other putative surface markers to isolate CSCs from tumors. Interestingly, CD44^+^/CD90^+^ cells [122] and CD44^+^/CD133^+^ [123] cells isolated from human HCC present a more aggressive phenotype than either CD133 positive or CD90 positive cells alone. Lee et al. revealed that CD44^+^ subpopulations higher self-renewal and circulating capacities than CD44^−^ compartment in a graft model [124]. In addition, CD133^+^ subpopulations preferentially expressed CD44 in four hepatic cancer cell lines, including Huh7, MHCC-97L, MHCC-LM3, and SMMC-7721. CD44^+^/CD133^+^ subpopulations exhibited high levels of stem cell-related markers and possessed a higher chemoresistance potential when compared to their CD44^−^/CD133^+^ counterparts [125]. CD44 down-regulation strongly suppressed tumor cell growth in vivo, increased apoptosis, and reduced chemoresistance in hepatic CSC subpopulations when compared with the control group [126]. Hence, there is an urgent need for developing potentially effective CD44-targeted therapeutic strategies. Cytoplasmic domains of CD44 can interact directly with various intracellular signaling molecules, including Src family kinases, GTPases, and adhesion molecules that regulate cell–cell interaction and motility [127,128]. CD44 interaction with HA can promote the growth, survival, migration, and invasion of cancer cells [129]. The binding of HA to CD44 promotes tumor cell growth in vivo by stimulating PI3K/Akt signaling pathway, which is known to stimulate cell survival [130]. Furthermore, CD44 also interact with various receptor tyrosine kinases (RTKs) [131], whose ligation has been implicated in cellular epithelial–mesenchymal transition (EMT) and metastasis [132]. CD44 variant isoform CD44v6 is involved in HCC cell growth by interacting with c-Met to stimulate RAS/MAPK signaling cascade [133]. Furthermore, its relationship with metastasis seems to be related to its role in the EMT [134,135].

### 4.5. CD90

Various CD markers have been served to identify CSCs in various HCC cell lines and primary clinical samples. CD90 (Thy-1) is a 25–37 kDa glycosyl phosphatidylinositol (GPI)-anchored membrane protein expressed mainly in leukocytes including hematopoietic stem and progenitor cells [136]. This glycoprotein is required for cell adhesion within a tissue. Recent studies demonstrated that the CD90^+^ cells showed a significant higher tumorigenic and metastatic potential than CD90^−^ counterparts when grafted to mice [137,138]. CD45^−^/CD90^+^ subpopulation derived from liver cancer specimens were showed higher self-renewal and tumor initiating potential than CD45^−^/CD90^−^ compartments [139,140]. Therefore, CD90 may also serve as a novel marker of poor prognosis and potential therapeutic target for the treatment of hepatocellular carcinoma. Yamashita et al. demonstrated that CD90 affects cell migration and invasiveness of EpCAM^+^ cells through the activation of TGF-β signaling pathway, whereas imatinib mesylate decreased CD90-induced cell migration of EpCAM^+^ cells by suppressing TGF-β expression [141]. Chen et al. also revealed that CD90 signal transduction through integrin-mTOR/AMPK-CD133 cascade is an important contributor to liver tumorigenesis [138]. Moreover, they also showed that inhibition CD90-mediated signaling pathway with a small-molecule agent OSU-CG5 significantly reduced the CD90^+^ cells in and subsequently repressed the liver cancer growth [138].

### 4.6. CD133

CD133 (also known as prominin-1) is a trans-membrane glycoprotein and an important cell surface marker for stem/progenitor cells in various types of tissue [142]. CD133-positive subpopulations of HCC cells were first reported as a potential CSC marker by Suetsugu et al. [143]. These authors found that the CD133-positive subpopulation showed a distinct high tumorigenicity in an immunodeficiency mouse xenograft model and lower levels of mature hepatocyte-specific markers, such as cytochrome P450 and glutamine synthetase (GS), when compared with CD133-negative counterparts [143]. Furthermore, Zhang et al. found that enhanced cytoplasmic CD133 expression is correlated with poor prognosis HCC patients [144]. Interestingly, normal stem/progenitor cells and CSCs share many features, including the capacity to self-renew and differentiate into multiple cell types [145]. A 70% partial hepatectomy (PHx) model in rodents has been widely used to study the precise regulatory mechanisms of self-renewal and differentiation in CSCs during tumor development that may also be related to liver homeostasis and regeneration. CD133 is significantly upregulated during early liver restoration following a strong regenerative stimulus, such as a 70% hepatectomy [146,147]. CD133 expression was significantly higher in a self-renewing subpopulation of human liver cancer cells, and it was absent in fully differentiated normal hepatocytes. Subsequent studies on CD133 expression in various human liver cell lines found the in vivo tumorigenic potential of these cells to positively correlate with CD133 expression [148]. Following a quantitative analysis of 41 HCC tissue specimens, Ma et al. found that CD133-positive cells were detected at low quantities in HCC (1.3–13.6% of the cells in the bulk tumor) [149], and Chen et al. also revealed that their presence negatively correlated with overall survival and recurrence rates [150]. The clinical significance of relative CD133 expression levels in HCC was similarly reported by Zhao et al. [123]. CD133 expressing stem-like HCC cell population has increased resistance to conventional chemotherapeutic agents by stimulating Akt signaling pathway [151]. Interestingly, Ma et al. demonstrated that miR-130b promotes CD133-positive tumor-initiating HCC cell growth and self-renewal ability by suppressing tumor protein 53-induced nuclear protein 1 (TP53INP1) expression [149]. Recently, Tang et al. provide in vitro and in vivo evidence that IL-8/CXCR1 signaling axis plays an important role in the self-renewal and angiogenesis in CD133 positive tumor-initiating HCC cells through the MAPK signaling pathway [152]. They also demonstrated that IL-8 levels were highly increased in CD133 positive cells isolated from HCC cell lines or clinical samples [152]. Additionally, Chen et al. demonstrate that the CD90-integrin-AMPK-CD133 signal cascade plays an important role in liver cancer [138]. These recent studies may lead to the development of more effective options against various types of hepatic cancer with high affinity and specificity.

## 5. Signaling Pathway-Based Therapies

### 5.1. Wnt/β-Catenin Signaling

The Wnt/β-catenin signaling is a highly conserved signaling pathway that regulates complex developmental and physiologic processes, including growth, regeneration, and self-renewal [153]. Aberrant activation of Wnt/β-catenin signaling is observed in approximately 30% of all HCCs, further emphasizing the important role of this signaling during hepatocarcinogenesis [154]. Recent studies have suggested that the expression of β-catenin, a key mediator of Wnt/β-catenin signaling, was significantly higher in HCC than its non-tumor counterparts [155]; in addition, inhibition of Wnt1-mediated signaling produced greater antitumor effects when compared with control groups [156]. Hepatoblastoma, a malignant embryonal tumor of the liver, was reported to be tightly linked to excessive Wnt/β-catenin signaling [157]. Furthermore, the interaction of Wnt/β-catenin signaling and CTNND1 markedly activates a specific gene transcriptional process that accelerates liver cancer progression and metastasis [158]. The incidence of activating mutations in β-catenin in HCC is as higher than p53 alterations [159]. Usually, aberrantly activated Wnt/β-catenin signaling can result in the abnormal stabilization of positive modulators of Wnt/β-catenin such as β-catenin or loss-of-function mutations in negative modulators of the signaling such as APC and Axin [160,161]. Consistently, loss-of-function mutations of many positive or negative regulators of the signaling such as TP53, AXIN (axis inhibition protein), and CTNNB1/β-catenin were observed in hepatocellular tumors [162,163]. Other well-known direct or indirect target genes of this signaling are CD44, C-Jun, Cyclin D1, C-Myc, VEGF, and MMP-7 [164,165]. Moreover, its target genes in the liver include cytochrome P450, EGFR, EpCAM, LECT2, glutamine synthetase, and SMP30 [166]. Moreover, Ji et al. found that activation of activated Wnt/β-catenin signaling enriched the EpCAM^+^ subpopulation by increasing the expression of four microRNA-181 family members [167]. These finding highlight the potential role of dysregulated Wnt/β-catenin signaling in the maintenance of stem-cell phenotypes and malignant liver cancer characteristics. In this context, various attempts have been made to develop various pharmacological inhibitors of the Wnt/β-catenin signaling. These inhibitors may help to eliminate hepatic CSC populations thought to be associated with multiple drug resistance, metastasis and tumor relapse.

### 5.2. Transforming Growth Factor (TGF)-β Signaling

TGF-β signaling plays a critical role in liver regeneration, but exerts growth-promoting effects in hepatic carcinogenesis via a number of effectors [168,169]. Importantly, HCC patients with raised TGF-β levels in serum samples had shown significantly lower survival rates when compared with patients who had normal TGF-β levels [170,171]. Increased levels of TGF-β are closely correlated with more advanced and aggressive tumor stages in HCC patients [172,173]. TGF-β signaling induces an EMT process in various types of liver cancer; however, the complex molecular mechanisms underlying this process are not fully understood [174]. A loss-of-function mutation in ELF, a Smad4 adaptor protein, was found to produce hepatocarcinogenesis through deregulated cell proliferation and promotion of tumor angiogenesis [175]. These studies suggest that TGF-β signaling can be a potential prognostic marker and therapeutic target in various types of hepatic cancer. The TGF-β signaling is comprised of three ligands (TGF-β1, TGF-β2, and TGF-β3) with different ligand binding affinities and signaling capabilities. TGF-β ligands bind to a single-pass transmembrane protein type II receptor, which in turn recruits and phosphorylates a second transmembrane kinase type I receptor. Upon ligand binding, the type I receptor phosphorylates the serine residue of the R-SMAD2/3 and results in ligand-induced transcription of various target genes [176,177]. SMAD7 is required for the down-regulation of TGF-β signaling by antagonizing activation of R-Smads [178]. Interestingly, TGF-β1 is known to promote the migration and EMT in HCC cells by enhancing snail expression and suppressing E-cadherin expression [179]. This process also seem to be affected by other ECM components by activating KAK-mediated Akt and ERK1/2 signaling [180].

### 5.3. Notch Signaling

The Notch signaling is a highly conserved signaling pathway that regulates complex developmental and physiologic processes, such as cell fate decisions and tissue pattern formations [181]. This signaling is also involved in the regulation of stem cell differentiation and maintenance [182,183]. Notch signaling activation occurs via an interaction between four Notch receptors (Notch 1–4) and five canonical ligands (Jag1, Jag2, Dll1, Dll3, and Dll4) [184,185]. It has been shown that Notch 1 and 2 share a similar basic structure and are ubiquitously expressed in a wide variety of tissues and cells types at varying levels [186,187]. In contrast, the expressions of Notch 3 and 4 are restricted to a more limited range of tissues and cell types, such as smooth muscle cells and vascular endothelial cells [188,189]. While the oncogenic functions of Notch signaling have been demonstrated in many human tumor types, its potential roles in the maintenance of CSCs have recently been identified in several solid tumors. Recent studies have suggested that suppression of Notch signaling markedly reduced the self-renewal potential and tumor-initiating capacity of colorectal CSCs [190]. Consistently, suppression of Notch signaling led to a greater decrease of the stem cells-like properties of and increased cellular sensitivity to ionizing radiation of glioblastoma-derived CSCs [191]. Aberrantly elevated Notch signaling is observed in CD133^+^ cells when compared with CD133^−^ compartments in HepG2 cell line [192]. Moreover, aberrant expression of Notch 3 and the notch ligand Jagged were recently observed in HCC [193,194]. Collectively, Notch signaling is as an important prognostic marker and could be used as a potential therapeutic target for hepatic cancers. Villanueva et al. have found that activation of Notch signaling may stimulate the HCC tumor formation in mice through direct activation of insulin-like growth factor 2 (IGF2) promotors [195]. They also revealed significantly enhanced expression of Notch signaling target genes such as Sox9, Hes1/2, Nrarp, and Spp1 in HCC compared them with four non-tumorigenic livers [195]. Moreover, Notch signaling target genes (e.g., DNASE1, CDK1, and CCND1/2) that are involved in cell cycle regulation were also significantly increased in HCC tissues as compared with non-cancerous liver tissue [195]. Zhou, et al. found that Notch signaling pathway inhibitor DAPT could suppress invasion of HCC cells via the ERK1/2 signaling pathways, resulting in the downregulation of MMP-2/9 and VEGF expressions [196].

### 5.4. Hedgehog Signaling

The Hedgehog (Hh), secreted glycoproteins, was first identified as a critical mediator of precise pattern formation during embryo development, and it is also involved in the regulation of growth, cell migration, and differentiation [197,198,199]. In mammals, Hh signaling is comprise of three known ligands, Sonic hedgehog (Shh), Desert hedgehog (Dhh), and Indian hedgehog (Ihh) [200]. Inappropriate activation of the Hh signaling has been described in a variety of human cancers including pancreatic, skin, and gastrointestinal cancers [201,202]. The Hh signaling cascade is initiated upon binding of the Hh ligand to the PTCH receptor and subsequent inhibition of the smoothened (SMO) [203]. Several research groups reported that Hh signaling is aberrantly activated in HCC [204,205,206]. Cai et al. demonstrated enhanced expression of Hh signaling components (PTCH1, Shh, Gli1) in liver cancer tissues compared with non-cancer tissues; in addition, these expressions positively correlated with tumor progression [206]. A recent study suggested that HCC cells secrete Shh ligands to induce glycolysis of adjacent glycolytic stromal cells, which consequently leads to the secretion of the lactate that HCC cells use as a source of energy [207]. Another study reported that the hepatic expression and activity of Hh ligands were significantly increased in all patients with chronic liver disease caused by the hepatitis C virus or HCC [208]. In addition, Hh signaling inhibition with synthetic small molecules reduces fibrosis [209]. Thus, hepatitis infections result in the increased production of Hh ligands and activated Hh signaling in liver cells, which in turn promotes liver cirrhosis and hepatocarcinogenesis. Consistently, aberrant activation of Hh target genes such as Gli1 and PTCH1 are observed in multiple types of HCC, indicating that the Hh signaling is frequently activated in HCC [210,211]. Wang et al. also demonstrated that suppression of Hh signaling significantly increased autophagy by up-regulating of Bnip3 (a member of BH3- only subset of the Bcl-2 family), which in turn stimulates apoptosis in various human HCC cell lines [212]. Patil et al. found that inhibiting the Hh pathway with GDC-0449, an Hh signaling antagonist, suppressed liver fibrosis and hepatocarcinogenesis in a murine model of primary liver cancer [209].

### 5.5. BMI1 Signaling

**Figure 3:**
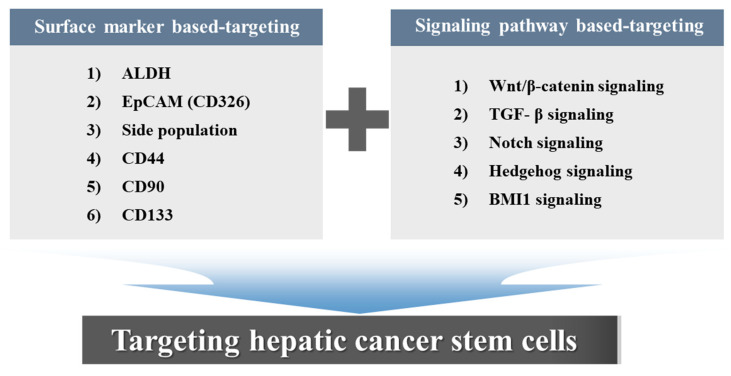
Schematic diagram summarizing the potential roles of stem cell-related markers or signaling pathways targeting hepatic cancer stem cells. Selectively targeting specific stem cell-related markers (aldehyde dehydrogenase (ALDH), EpCAM, side population, CD44, CD90, and CD133) and/or -signaling pathways (Wnt/β-catenin, TGF-β, Notch, Hedgehog, and BMI1 signaling) of hepatic CSCs is an effective therapeutic strategy for treating chemotherapy-resistant liver cancer.

BMI1, also known as polycomb group RING finger protein 4, is a highly conserved regulatory factor throughout evolution [213]. BMI1 functions as an epigenetic regulator of gene expression and is also known for its significant influence on embryonic development at different stages of life and stem cell self-renewal and differentiation [214,215]. Recently, BMI1overexpression has been found in various types of cancer and is associated with the poor overall survival of patients [216,217,218]. Consistently, BMI1 overexpression is highly correlated with malignant phenotypes and thereby causes malignant transformation in HCC [219,220,221]. Aberrant BMI1 expression contributes to the maintenance of CSC subpopulations in multiple types of cancer [34,222,223,224]. Additionally, BMI1 is involved in the maintenance of the tumorigenic SP subpopulation in liver cancer [113]. BMI1 was first identified as an oncogenic effector for the development of lymphocytic leukemia by suppressing c-Myc, which is dysregulated in multiple cancer types, such as colon, lung and liver cancers [225,226,227]. The inhibition of the Ink4a/Arf locus-specific binding of BMI1 reduced cell proliferation and increased senescence of stem cells or CSCs [228,229]. BMI1 gene silencing in the HCC cells inhibited sphere formation ability in vitro and tumorigenesis in vivo by blocking the cell cycle transition from the G0/G1 to the S phase [230]. Xu et al. found that while activating BMI1 or RasV12 alone was insufficient to enhance HCC development, over-expression of these two genes at the same time is suspected to promote tumor formation in mice [231].This results suggested that BMI1 can cooperate with other oncogenes to stimulate hepatocarcinogenesis in vitro and in vivo. Effendi et al. demonstrated that the suppression of BMI1 expression was followed by a respective decrease in drug efflux protein ATP-binding cassette transporter B1 (ABCB1) expression [232]. BMI1 signaling was highly activated in CD133^+^ liver CSCs and plays an important role in the maintenance of liver hepatic stem/progenitor cells in mice [233]. Furthermore, BMI1 knockdown drastically reduced the number of SP cells, and the knockdown of BMI1 in SP cells significantly abolished their tumorigenicity in HCC [113,146,234]. Collectively, BMI1 may be a potential target for the diagnosis and therapy of hepatic cancers. The potential roles of stem cell-related markers or signaling pathways targeting hepatic cancer stem cells are summarized in Figure 3.

## 6. Potential Clinical Application of Liver Cancer Stem Cells

### 6.1. Aspects of Diagnosis and Prognosis

Although great advances in diagnosis and therapeutic strategies for liver cancer have remarkably enhanced the chances for successful treatment at early stages, 30–60% of patients relapse after conventional therapy [235]. New diagnostic approaches selectively with specific hepatic CSC markers are getting more attention for evaluating the tumor progression and therapeutic effects [7]. As previously mentioned, CD133 expression is negatively correlated with overall survival and recurrence rates in patients with HCC [144,236,237,238]. HCC patients with higher level of CD133 expression in primary lesion have shorter 5-year survival times and higher relapse rates after surgical resection compared to patients with lower CD133 expression [144,239]. The up-regulated expression levels of CD133 have also been related to the ability to survive under hypoxia or malnutrition by performing autophagy [240]. HCC patients with higher level of CD133 expression have also shown poorer response to conventional chemotherapeutic agent Sorafenib [241]. It has been shown that the CD90^+^ cells showed a significant higher tumorigenic and metastatic potential than CD90^−^ counterparts, suggesting its role as a marker for metastatic liver cancer [242,243]. Consistent with these findings, CD90 also affects cell migration and invasiveness of EpCAM^+^ hepatic CSCs through the activation of TGF-β signaling pathway [141]. Thakolwiboon et al. also demonstrated a correlation positive between CD90 expression levels and tumor progression in hepatocellular carcinoma [244]. Moreover, Lu et al. revealed that HBV infection, one of the leading causes HCC, significantly increased CD90 expression and its high expression was positively correlated with poor prognosis [245]. Therefore, CD90 may also serve as a novel marker of poor prognosis and potential therapeutic target for the treatment of hepatocellular carcinoma. CD44 expression is associated with metastatic phenotype [246] and CD44 variant isoform CD44v6 is involved in HCC cell growth by interacting with c-Met to stimulate RAS/MAPK signaling cascade [133]. Furthermore, its relationship with metastasis seems to be related to its role in the EMT [247,248,249]. Interestingly, CD44^+^/CD90^+^ cells [243] and CD44^+^/CD133^+^ [125] cells isolated from human HCC present a more aggressive phenotype than either CD133 positive or CD90 positive cells alone. These double positive cells exhibited enhanced tumorigenicity and chemoresistance probably via the increased expression of stemness-related genes [125]. Hence, CD44 may also serve as a novel marker of poor prognosis and potential therapeutic target for the treatment of HCC. Taken together, these studies suggest that the these characteristics of hepatic CSCs can be useful to predict tumor progression and survival of patients with HCC, although further studies are warranted to fully elucidate their impact on prognosis and treatment.

### 6.2. Liver CSCs-Targeted Therapy

**Figure 4:**
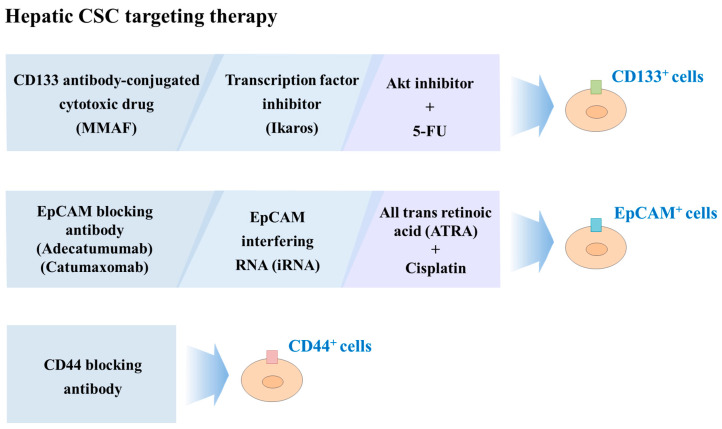
Schematic diagram summarizing the effect of target therapy on hepatic CSCs. CD133 antibody conjugated to a cytotoxic drug (monomethyl auristatin F), transcription factor inhibitor Ikaros, and Akt signaling inhibitor with 5-FU reduced the self-renewal capacity of CD133+ hepatic CSCs. All-trans retinoic acid (ATRA) with cisplatin, Oncostatin M (OSM), EpCAM-blocking antibodies (Adecatumumab and Catumaxomab), and EpCAM blocking RNA interference (RNAi) inhibited the self-renewal capacity of CD133^+^ hepatic CSCs. In addition, anti-CD44 antibody prevented the tumor formation and metastasis of CD44-positive hepatic CSCs.

CD133 is one of mostly defined and well-characterized hepatic CSC markers, thus, many investigators have tried to develop therapeutic strategies targeting CD133^+^ cells. Smith et al. demonstrated that CD133 antibody conjugated to a cytotoxic drug (monomethyl auristatin F) significantly enhance the antitumor effects and reduce adverse systemic effects of potent cytotoxic drugs in hepatocellular and gastric cancers [250]. Zhang et al. found inhibitory effects of transcription factor Ikaros on the self-renewal capacity of CD133^+^ hepatic CSCs via direct binding to the CD133 P1 promoter and subsequent suppression CD133 expression [251]. Similarly, a murine anti-human CD133 antibody (AC133) conjugated to a potent chemotherapeutic agent, monomethyl auristatin F (MMAF), significantly reduced the self-renewal capacity of CD133^+^ hepatic CSCs and subsequently delayed tumor formation in a SCID mouse model [250]. Blocking the Akt signaling pathway can inhibit the growth of CD133-positive HCC cells and sensitize to chemotherapeutic drug 5-FU [76]. Zhang et al. demonstrated that all-trans retinoic acid (ATRA) could induce differentiation of EpCAM^+^ HCC CSCs to decrease their tumorigenic potential by reducing CSC-related markers and increasing hepatocyte-specific genes. Consistent with this result, the efficacy of the combinational therapy of cisplatin and ATRA was more prominent than either drug treatment alone [252]. Yamashita et al. found that Oncostatin M (OSM) resulted in the reduction of tumorigenic capacity by inducing the differentiation of EpCAM^+^ hepatic CSCs. Moreover, the combinational therapy of 5-fluorouracil and OSM synergistically suppressed HCC by targeting both CSCs and non-CSC cells [253]. The CD90/CD44 double positive cells exhibited more tumorigenic phenotypes than the CD90/CD44 double negative counterpart [243]. Indeed, CD44 blockade with anti-CD44 antibody prevented the tumor formation and metastasis of CD44-positive hepatic CSCs in vivo [243]. Currently, several EpCAM-blocking antibodies are in clinical development, such as Adecatumumab (recombinant human IgG1 monoclonal antibody) and Catumaxomab (trifunctional IgG2 antibody). In liver cells, RNA interference-based blockage of EpCAM significantly inhibited the self-renewal and differentiation capacity of hepatic cancer stem-like cells in vitro and in vivo [102]. Taken together, these findings suggested that therapeutic strategy that focuses on targeting CSCs can be an alternative approach to overcome the limitations of traditional liver cancer treatments. The potential effects of target therapy on hepatic CSCs are summarized in Figure 4.

## 7. Future Perspectives

Primary liver cancer consists predominantly of HCC and ICC. Liver CSCs may be the origin of some HCCs and ICCs. This CSC hypothesis explains why only a minority of the cells from most liver cancers with malignant phenotypes are clonogenic in vitro and in vivo. However, several critical questions on liver cancer development remain to be addressed, including the origin of liver CSCs, whether CSCs originate from normal stem cells or from more differentiated progenitor cells, the effect of hepatitis virus infection, the functional involvement of the liver CSC niche, and the cause of CSC emergence [254]. The most of the currently available knowledge about hepatic CSCs is largely influenced by the basic biological features of normal stem cells such as distinct signaling pathways and/or surface markers. In this context, targeting these common biological characteristics to eliminate hepatic CSCs may reduce normal hepatic stem cells and subsequently prevent the normal liver regeneration. Until now, it remains unclear whether HCC CSCs can be selectively depleted without unduly affecting normal and healthy liver stem cells. Therefore, further characteristics associated with CSC-specific cell surface markers and signaling pathways need to be investigated.
